# Commencement Exercises

**Published:** 1902-07

**Authors:** 


### COMMENCEMENT EXERCISES.

### VETERINARY DEPARTMENT, UNIVERSITY OF PENNSYLVANIA.

The one hundred and forty-sixth annual commencement exercises of this department was held on June 18th, at 11 A.M., in the Academy of Music. The exercises were participated in by all the departments of the University. After invocation and hymn, "Our Father in Heaven," the orator of the day, Dr. Nicholas Murray Butler gave an excellent address. Singing of hymn, "Hail Pennsylvania," then conferring of degrees. There were twenty-two graduates—Sumner C. Babson, Gloucester, Mass.; Howard Baker, Dudley, Mass.; Harry C. Bassler, Philadelphia, Pa.; Fred C. Bigelow, Philadelphia, Pa.; F. Henry Bradley, Newport, R. I.; Samuel Burrows, Cleveland, Ohio; Guy T. Cole, Norwalk, Ohio; Herman H. Delano, North Duxbury, Mass.; Hyma Feigenbaum, Pittsburg, Pa.; Frank U. Fernsler, Lebanon, Pa.; William H. Glass, Philadelphia, Pa.; Arthur A. Harmon, Chelmsford, Mass.; Joseph A. McClintock, Philadelphia, Pa.; Anthony J. McCloskey, Philadelphia, Pa.; George L. Nicholas, South Bethlehem, Pa.; Kenneth E. Paget, Scranton, Pa.; Robert O. Rothermel, Reading, Pa.; Oscar F. Stearns, Philadelphia, Pa.; Joseph W. Vansant, Hulmeville, Pa.; Frederick Weitzel, Allegheny, Pa.; Benjamin T. Woodward, Philadelphia, Pa.; John H. Zollinger, Philadelphia, Pa.

Singing of hymn, "Now Thank We Our God;" Benediction.

The regular annual meeting of the Alumni Society was held in Houston Hall, followed by a smoker in place of a banquet.

---

Veterinary Surgeon Desmond, of Adelaide, South Australia, has reported upon several new conditions among domesticated animals and poultry in that country. Actinomycosis has been noted and reported upon. Among other unusual cases was an endothelioma in an aged stallion. The tumor weighed thirty pounds. Leukemia in poultry from a specimen exposed for sale in the open market is also reported upon.

